# Identification of Competing Endogenous RNA and Micro-RNA Profiles and Regulatory Networks in 4-Nonylphenol-induced Impairment of Sertoli Cells

**DOI:** 10.3389/fphar.2021.644204

**Published:** 2021-05-18

**Authors:** Wenjie Liu, Zhaokai Wang, Xiaopeng Hu

**Affiliations:** ^1^ School of Pharmaceutical Sciences, Fujian Provincial Key Laboratory of Innovative Drug Target Research, Xiamen University, Xiamen, China; ^2^ Technical Innovation Center for Utilization of Marine Biological Resources, Third Institute of Oceanography, Ministry of Natural Resources, Xiamen, China; ^3^ Bio-X Institutes, Shanghai Jiao Tong University, Shanghai, China

**Keywords:** 4-nonylphenol, androgen receptor, sertoli cell, circRNA, ceRNA

## Abstract

The xenoestrogens nonylphenols (NPs), which are materials used in the plastic polymer industry, are considered endocrine disruptors in a wide range of organisms. Studies have shown that human health problems, such as infertility and reproductive toxicology, are linked with NPs. However, the mechanism by which NPs interfere with male reproduction is not fully elucidated. Here, we found that 4-NP can result in male reproductive impairment and reduce androgen receptor (AR) protein levels in rat sertoli cells *in vitro* and *in vivo*. Moreover, we performed RNA sequencing to assess the differential expression of ceRNAs in rat primary sertoli cells treated with 4-NP. Bioinformatics methods, such as Gene Ontology (GO), Kyoto Encyclopedia of Genes and Genomes (KEGG) database and ceRNA functional network analyses, were used to investigate the sequencing data and gain further understanding of the biological processes. Our analysis revealed a core set of mRNAs (*Ar*, *Atf6* and *Cbp*), and circRNAs (*circ673*, *circ1377*, *circ1789*, and *circPTEN*) that were selected and validated by RT-qPCR. In addition, the head-to-tail splicing of *circ673*, *circ1377*, *circ1789*, and *circPTEN* was identified by Sanger sequencing. These findings provide the first insight into the ceRNA expression profiles of rat sertoli cells and reveal that ceRNAs participate in 4-NP-induced impairment of sertoli cell function, thereby indicating potential therapies for both reproductive toxicology and male infertility.

## Introduction

Nonylphenols (NPs) are some of the most widely used synthetic xenoestrogens in laundry and dish detergents, textiles, plastics, pesticides, paper and personal care products. Considering that NPs are so widely used because of their numerous applications, humans are unavoidably exposed, most likely throughout their lifetimes. A study by the Centers for Disease Control and Prevention (CDC) detected NPs in the urine of >50% of Americans sampled in 2005 (Calafat et al., 2005). Another previous study showed that NPs can induce changes in the metabolism and secretion of endogenous hormones and increase the risk of numerous pathologies, including prostate cancer. Recently, NPs have attracted considerable attention due to their estrogenic potential as well as their ability to induce the development of diabetes Yu et al. (2018), cancer Forte et al. (2016), Gan et al. (2014) and reproductive diseases (El-Dakdoky and Helal, 2007). Moreover, adverse effects of NPs on male reproductive function have been demonstrated in fish Ishibashi et al. (2006), ducks Cheng et al. (2016), mice Jambor et al. (2017), rats Kazemi et al. (2016) and humans.

Previous reports have shown that sperm counts in men have dropped by 50% over the last half-century. The intense ongoing drop in sperm count has been linked to a wide range of factors, such as exposure to certain chemicals. Some man-made compounds, known as “environmental hormones” or xenoestrogens, are considered to mimic the behavior of natural estrogen in the body and result in inappropriate programming of the reproductive system. Such compounds have traditionally been blamed for the recession in male reproductive health and fertility. NPs are classical xenoestrogens with long-lasting toxicity that are harmful to the reproductive systems of animals and humans. It is well known that there is a balance of sex hormones in the body that controls multiple aspects of reproductive development. However, how xenoestrogens, including NPs, cause endocrine disruption is unclear.

Spermatogenesis and male fertility are dependent upon sertoli cells (Dubois and Callard (1989), Hu et al. (2015), Johnson et al. (2008), Shinohara et al. (2003), which provide nourishment and mechanical support to germ cells (Rich and de Kretser, 1983). Every sertoli cell supports only a certain number of germ cells (Berndtson and Thompson, 1990; Johnson et al., 2008). sertoli cells also synthesize and secrete some functional products that are important for support and nourishment of germ cells and for initiation of spermatogenesis (Dubois and Callard, 1989; Alves et al., 2013). Several studies have reported that NPs induce apoptosis, autophagy and necrosis in sertoli cells (Choi et al., 2014; Duan et al., 2016; Duan et al., 2017).

Previous studies have revealed that androgen receptor (AR) is not expressed in germ cells (GCs) but is expressed in Leydig cells (LCs), myoid cells (MCs) and sertoli cells (SCs) (De Gendt and Verhoeven, 2012). sertoli cell AR (SCARs) is critical for regulation of testicular development and for ongoing maintenance of spermatogenesis Holdcraft and Braun (2004), and disruption of AR in SCs leads to meiosis arrest and male infertility in mice (Chang et al., 2004; De Gendt et al., 2004; Lim et al., 2009). NPs have been interpreted to mimic the natural sex hormone estradiol given their binding affinity for AR, estrogen receptors (ERα and ERβ), peroxisome proliferator-activated receptors (PPARs) and aryl hydrocarbon receptors (AHRs) (Laws et al., 2000; Cavanagh et al., 2018). NPs have also been reported to affect the expression of cell receptors in sertoli TM4 cells of mice, and the mRNA expression of AR is downregulated after NP exposure (Liu et al., 2014).

Studies have shown that circular RNAs (circRNAs) and competing endogenous RNAs (ceRNAs) are involved in testis development and spermatogenesis (Dong et al., 2016; Ge et al., 2019; Meng et al., 2019; Jia et al., 2020). ceRNAs include all RNA transcripts, including protein-coding messenger RNAs (mRNAs) and non-coding RNAs, such as long noncoding RNAs (lncRNAs), circRNAs and pseudogenes, that can modulate each other by competing for shared microRNAs (miRNAs) (Qu et al., 2015). circRNAs and lncRNAs have an adsorptive effect on miRNAs, acting as miRNA "sponges" that naturally isolate and competitively inhibit miRNA activity (Qu et al., 2015). It has been reported that NPs exert effects Jubendradass et al. (2012) by regulating multiple signaling pathways; for example, they can change DNA methylation and histone modification (Jubendradass et al., 2012; Ghosh et al., 2019; Kim et al., 2019). NPs also induce apoptosis, autophagy and DNA damage *via* activation and modification of Poly ADP-Ribose Polymerase (PARP) and Histone H2AX in human keratinocytes (Kim et al., 2018). Several studies have reported that NPs induce apoptosis, autophagy and necrosis in sertoli cells *via* multiple signaling pathways (Han et al., 2004; Gong et al., 2009; Choi et al., 2014; Duan et al., 2016; Huang et al., 2016; Duan et al., 2017). However, the underlying ceRNA mechanism has not been clarified.

Here, we explored the expression of AR and ceRNAs, which may be relevant for 4-NP-induced reproductive impairment, and investigated ceRNA crosstalk (for example, with mRNAs, miRNAs, circRNAs, and lncRNAs) by strand-specific RNA sequencing.

## Materials and Methods

### Animals and Treatments

Male rats (6–9 weeks old, Sprague-Dawley) were purchased and allowed to acclimate for approximately one week before being used in the study. The animals were subjected to 12 h/12 h light/dark cycles in temperature-controlled rooms. Water and food were provided ad libitum. 4-Nonylphenol (100 mg/kg, Sigma-Aldrich) was dissolved in olive oil and then applied by (ip) injection, while the equivalent volume of vehicle alone was applied as a control. 4-Nonylphenol exposure was continued for 14 days. The rats were used for the experiments according to the animal care guidelines, and all animal procedures were approved by the Institutional Laboratory Animal Use and Care Committee of Xiamen University.

### Paraffin Sections and Hematoxylin Eosin Staining

Testicular samples were collected and incubated in 4% paraformaldehyde (PFA) for fixation on a rotator overnight at 4°C and subsequently embedded and sectioned using a rotary microtome (Leica). For hematoxylin eosin (HE) staining, the sections were deparaffinized in xylene, rehydrated in a graded series of ethanol washes, washed with tap water, and then stained with hematoxylin and eosin.

### Immunofluorescence Staining

For immunofluorescence staining, the sections were deparaffinized in xylene, rehydrated in a graded series of ethanol washes and washed with PBS. Then, antigen retrieval was performed in a microwave oven for 20 min in antigen-unmasking solution. The activity of endogenous peroxidase was blocked by 3% hydrogen peroxide in methanol, and nonspecific staining was blocked with a BSA solution for 30 min. The testicular sections were incubated with a primary antibody against androgen receptor (AR, Santa Cruz) overnight at 4°C and subsequently washed three times in PBS. Then, an IgG-Alexa 594 (Invitrogen-Molecular Probes) secondary antibody was applied for 1 h at 37°C. The sections were mounted in antifade fluorescence mounting medium with DAPI (Invitrogen). The slides were imaged using an Olympus microscope (BX51) with a digital camera.

### Primary Sertoli Cell Culture and Treatments

Isolation and culture of primary sertoli cells were performed as described in a previous study (Hu et al., 2015). The testes were harvested from rats (5–10-day-old Sprague-Dawley rats) and processed for sertoli cell enrichment. Briefly, the testes were sliced using a razor blade into fine (∼1 mm wide) pieces and digested with trypsin (1 mg/ml, Gibco) and DNase I (0.8 mg/ml, Sigma-Aldrich) in F12/DMEM for 20 min at 37°C. Then, the dissociated cells were passed through a 100 µm filter and subjected to centrifugation at 800 × g for 5 min, and the pellets were resuspended in 40 ml of a solution containing 0.01% soybean trypsin inhibitor, 0.8 mg/ml dnase I, 2 mM EDTA, and 1 M glycine for 10 min. The samples were then centrifuged at 800 × g for 5 min and collected. The collected samples were then suspended and further digested in a solution of collagenase I (Sigma-Aldrich, 1 mg/ml), DNase I (Sigma-Aldrich, 0.8 mg/ml) and hyaluronidase III (Sigma-Aldrich, 1 mg/ml) at 37°C for 20 min. The cells were then centrifuged at 800 × g for 5 min and collected. The resulting cells were cultured in F12/DMEM (1:1) containing 10% fetal bovine serum (FBS). Primary sertoli cells (P2) were treated with 0, 0.2, 0.5, and 1 mM 4-nonylphenol (Sigma-Aldrich) separately in a humidified incubator under 5% CO_2_ at 37°C.

### Western Blot Analysis

After treatment, sertoli cells were collected, and the protein content of the supernatant was detected with a Thermo Scientific Pierce BCA Protein Assay Kit. The protein samples were run and separated by SDS polyacrylamide gel electrophoresis. After electrophoresis, a Trans-Blot system (Bio-Rad) was used to transfer the proteins to PVDF membranes at 90 V for approximately 1–2 h. The blots were blocked with 5% (w/v) nonfat skimmed milk for 60 min at room temperature. Then, the membranes were incubated with primary antibodies against androgen receptor (AR, Santa Cruz) overnight at 4°C. Subsequently, horseradish peroxidase (HRP)-conjugated secondary antibodies were added, and the blots were incubated for 60 min at room temperature. The results were detected with enhanced chemiluminescence (ECL) solution. The immunoblots were finally photographed using a Bio-Rad image analyzer.

### RNA Isolation

Total RNA was extracted from 4-NP-treated primary sertoli cells for bulk RNA sequencing using an RNA extraction kit (TaKaRa, Dalian, China), and the A260/A280 values were quantified in the range of 1.8–2.1. Quality control of the RNA was performed, and the RNA met the sequencing requirements. Raw sequencing data have been submitted to the NCBI Sequence Read Archive under accession number GSE167912.

### Computational Analysis of ceRNAs

Quality control of the raw reads was performed with a standard procedure and verified with FastQC (http://www.bioinformatics.babraham.ac.uk/projects/fastqc/) in this study. We used the R package edgeR to analyze the differentially expressed ceRNAs, and ceRNAs with log2 values > 1 or log2 values < −1 were selected as significantly differentially expressed (*p* < 0.05).

### GO and KEGG Enrichment Analyses

To further perform bioinformatics analysis, the Gene Ontology (GO) and Kyoto Encyclopedia of Genes and Genomes (KEGG) databases were used to analyze the differentially expressed circRNA cognate mRNAs, lncRNA targets and mRNAs. GO terms and KEGG pathways with corrected *p* values <0.05 were significantly enriched.

### Quantitative PCR Analysis

Tissue was immediately saturated in RNAlater until analysis. Total RNA from sertoli cells was reverse-transcribed into cDNA with a HiScript Reverse transcriptase Kit (Vazyme). Quantitative PCR was performed with Fast Start universal SYBR Green Master Mix (Roche) in a Real-Time PCR System (7,500, ABI). *Gapdh* transcript levels were used to normalize the relative expression of the target genes. The primer pairs (circRNAs and mRNAs) for quantitative PCR are shown in Supplementary Table S1.

### Prediction of miRNA Targets and Construction of a ceRNA Network

The 3’ untranslated regions (UTRs) of mRNAs, circRNAs and lncRNAs were used to predict miRNA targets with miRanda and TargetScan. As circRNAs and lncRNAs contain multiple miRNA binding sites, both are considered ceRNAs that bind to miRNA competitively and indirectly influence mRNA expression. A network of ceRNA interactions, such as lncRNA/miRNA/mRNA and circRNA/miRNA/mRNA interactions, was constructed with local Perl scripts.

### Statistical Analysis

The data analyses were performed with GraphPad Prism 7. Probability values of < 0.05 were considered to indicate statistical significance.

## Results

### Morphological Alterations of Testes in 4-NP-Treated Rat

**FIGURE 1:**
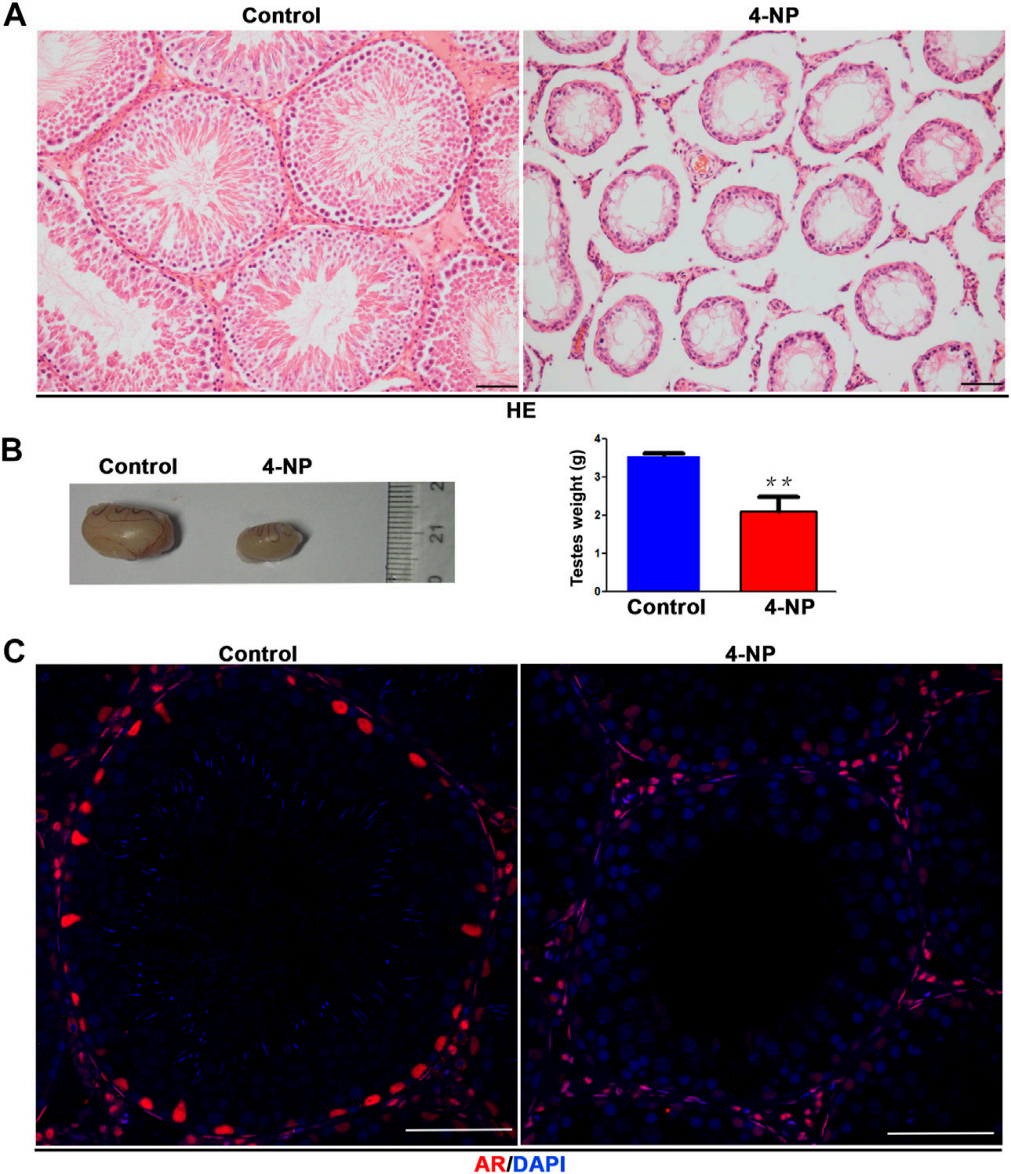
4-NP has significant male reproductive toxicity and can reduce the protein expression of androgen receptor (AR) in sertoli cells of rat. **(A)** Histological examinations of 4-NP-treated (100 mg/kg/day; for 14 days) rats showed damage and arrest of spermatogenesis. Scale bar, 50 μm. **(B)** 4-NP (100 mg/kg/day; for 14 days) reduced the sizes and weights of rat testes. The values are expressed as the mean ± standard error of the mean (SEM) for Eight rats. Statistical analysis was performed using Student’s t-test. Asterisks denote statistical significance; **p < 0.01, ***p < 0.001. **(C)** Immunofluorescence staining of the AR protein in seminiferous tubules of 4-NP-treated (100 mg/kg/day; for 14 days) rats. The number of AR-positive cells in per tubule (cross section) in section of each testis tissue (Eight 4-NPs, Eight controls, Ten tissue sections per testis) was counted. Statistical analysis was performed using Student’s t-test. Scale bar, 50 μm.

The morphology of 100 mg/kg/day 4-NP-treated (for 14 days) rat testes groups was examined by HE staining. Histological examinations of 4-NP-treated rats showed severe damage and arrest of spermatogenesis (Figure 1A) and testes that were significantly smaller than those in the control group (Figure 1B). Compared with the seminiferous tubules in the control group, which contained the full complement of germ cells, those in the 4-NP-treated group had smaller diameters, and only morphologically normal sertoli cells and undifferentiated spermatogonia cells were observed in the 4-NP-treated group (Figure 1A) and the seminiferous epithelium was also completely lacking in differentiated germ cells; at the basal membrane, only sertoli cells and undifferentiated spermatogonia were observed (Figure 1A). Moreover, although immunofluorescence analysis revealed that the signal intensity of AR in 4-NP-treated groups was weaker than that of the controls, the numbers of AR-positive cells (sertoli cells) in tubules were not significantly lower in 4-NP-treated rat testes than in control testes *in vivo* (Figure 1C). This result suggested that 4-NP maybe decreased the expression of AR and impair the function of sertoli cells.

### 4-NP Exposure Decreases the Expression of Testicular AR

**FIGURE 2:**
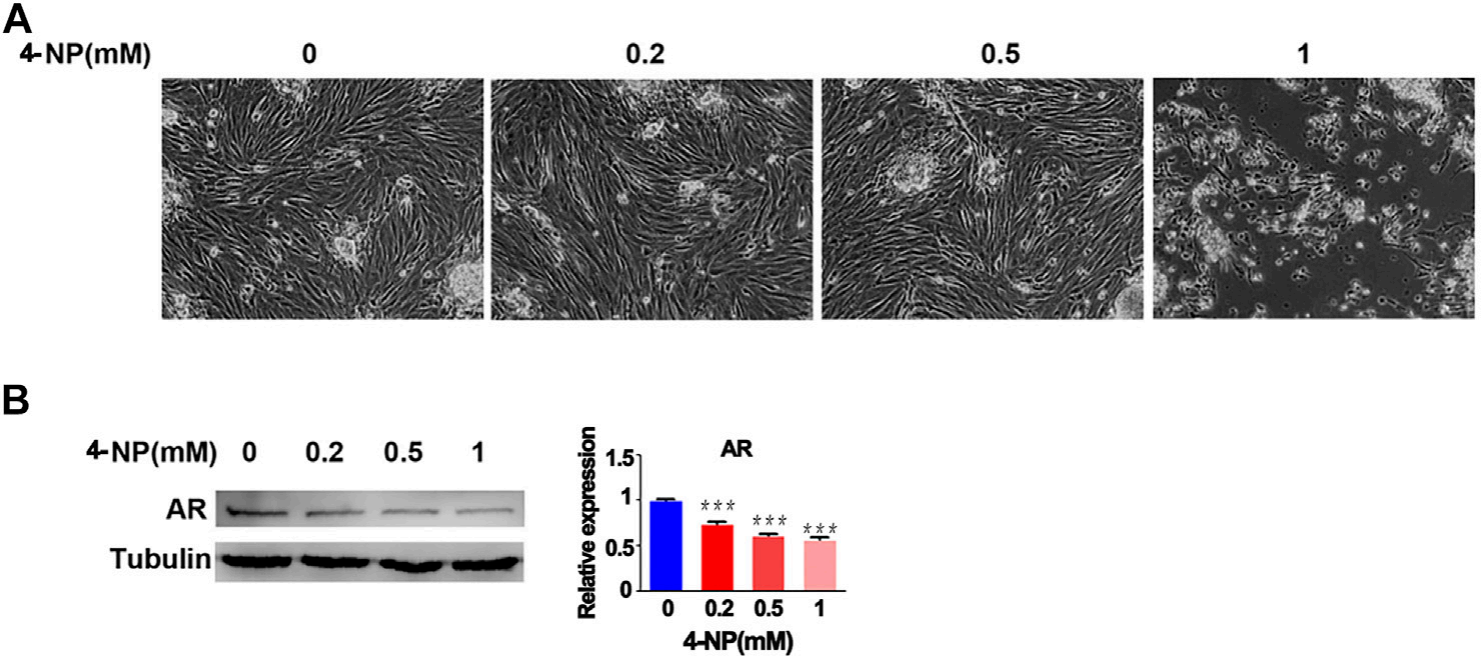
4-NP treatment of rat sertoli cells *in vitro*. Morphological alterations in sertoli cells exposed to differing concentrations of 4-NP **(A)** and change in the numbers of sertoli cells exposed to differing concentrations of 4-NP **(B)**. Whole cell extracts were prepared and analyzed by western blotting with antibodies against AR and loading control Tubulin. The values are expressed as the mean ± standard error of the mean (SEM). Statistical analysis was performed using Student’s t-test. Asterisks denote statistical significance; **p < 0.01, ***p < 0.001.

Then, to evaluate whether 4-NP impaired the function of sertoli cells and reduced the expression of AR, we investigated the effect of 4-NP on rat primary sertoli cells *in vitro* and treated the cells with differing concentrations of 4-NP (0, 0.2, 0.5, and 1 mM) at 24 h. When compared with no 4-NP-treated rat primary sertoli cells, there were no morphological alterations in sertoli cells exposed to 4-NP (0.2 and 0.5 mM) and no apparent change in the numbers of sertoli cells exposed to 4-NP (0.2 and 0.5 mM), but cell death and decreased numbers of cells were observed in 1 mM 4-NP-treated group (Figure 2A). Furthermore, we investigated the changes in the protein levels of AR in primary sertoli cells associated with 4-NP exposure. Sertoli cells were exposed to 4-NP (0, 0.2, 0.5, and 1 mM), and the AR levels in the sertoli cells were determined by western blot analysis (Figure 2B). The protein levels of AR were significantly lower in the 0.2, 0.5, and 1 mM 4-NP-treated cells than in the control cells (Figure 2B). These findings revealed that 4-NP reduced AR protein levels in rat sertoli cells and impaired sertoli cell function.

### Analysis of Differentially Expressed miRNAs, CircRNAs, lncRNAs and mRNAs in 4-NP-Treated Rat Primary Sertoli Cells *in vitro*

To uncover the ceRNA mechanism underlying the effect of 4-NP on sertoli cells *in vitro*, systematic ceRNA/small RNA sequencing (ceRNA/small RNA-Seq) was performed. Raw sequencing data have been submitted to the NCBI Sequence Read Archive under accession number GSE167912. We included three controls and three 4-NP (0.5 mM)-treated sertoli cell samples in the miRNA-Seq experiment, and we included two controls and two 4-NP (0.5 mM)-treated sertoli cell samples in the ceRNA-Seq experiment. The ceRNA/miRNA-Seq data were analyzed by bioinformatics methods. Strict filtering methods were used to compare the 4-NP group and the control group (fold change: >2 or < 0.5; *p* < 0.05) (Supplementary Figures S1A, S2A, S3A, S4A). We identified 54 upregulated and 82 downregulated miRNAs (Supplementary Figure S1B), 465 upregulated and 419 downregulated circRNAs (Supplementary Figure S2B), 74 upregulated and 191 downregulated lncRNAs (Supplementary Figure S3B), and 1,747 upregulated and 2,659 downregulated mRNAs (Supplementary Figure S4B).

FastQC quality control was performed. A Q-score higher than 10 (error rate < 10%) was required for each replicate for circRNAs (Supplementary Figure S2D) and lncRNAs (Supplementary Figure S3E). The variability of the ceRNA sequences in 4-NP-treated samples relative to controls is displayed in box plots (Supplementary Figures S2C, S3C, S4C). In addition, the correlation coefficients of the replicated samples had good consistency (Supplementary Figures S1C, S3D).

**FIGURE 3:**
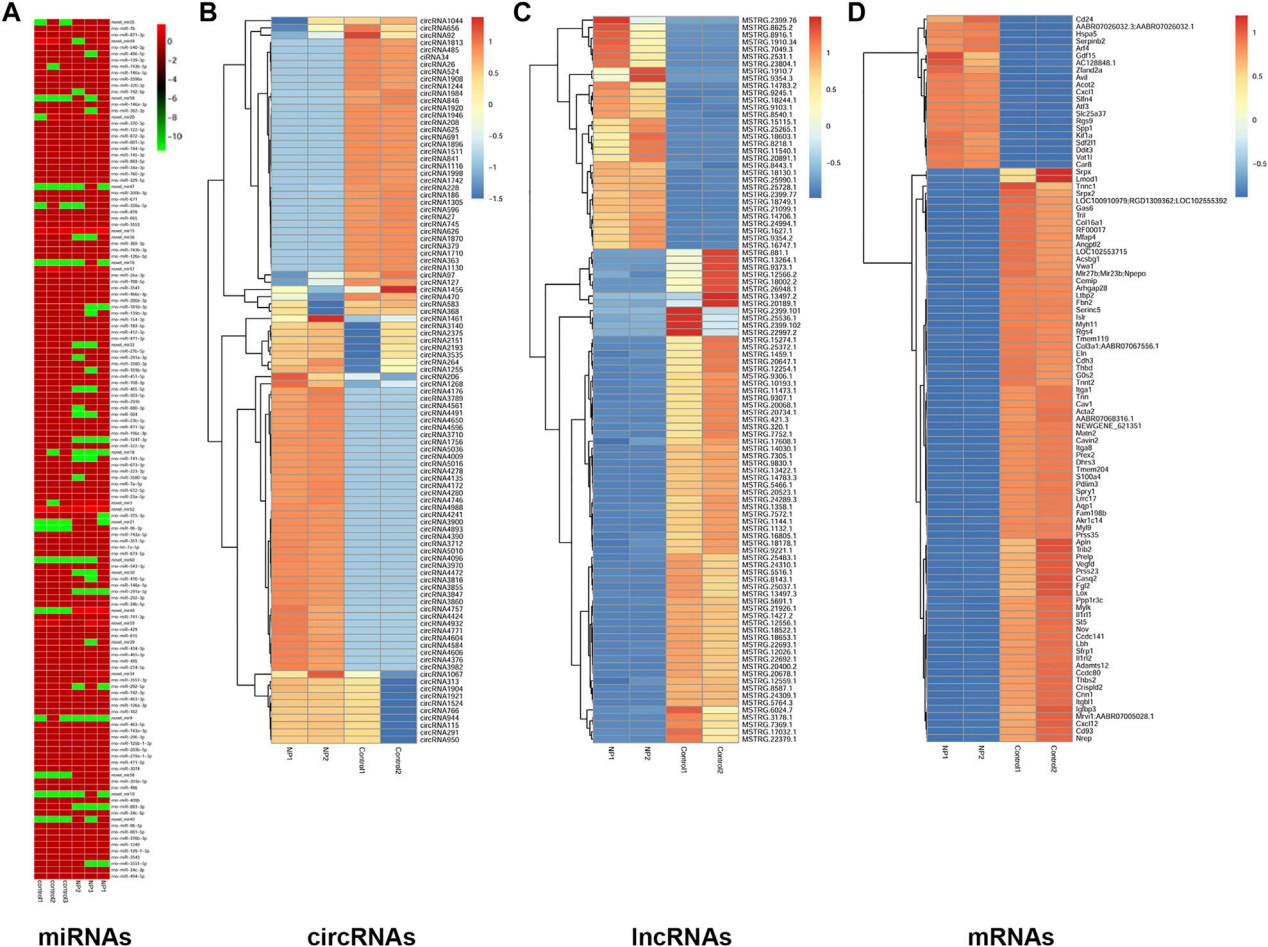
Hierarchical clustering of miRNAs **(A)**, circRNAs **(B)**, lncRNAs **(C)**, and mRNAs **(D)** in rat sertoli cells between 4-NP (0.5 mM)-treated rats and controls. The color bar presents the expression level of miRNA.

For miRNAs and ceRNAs. The length distribution of small RNAs is shown in Supplementary Figure S1D, and the precision of the RPKM analysis for the mRNA-Seq experiments is shown in Supplementary Figure S4D. Moreover, based on the differentially expressed miRNAs (Figure 3A), circRNAs, lncRNAs, and mRNAs, a tree was generated by MeV_4_9_0 cluster analysis. The tree clearly separated 4-NP-treated samples from controls (Figures 3B–D). These results indicated that ceRNA expression could be used to robustly separate control and 4-NP-treated samples.

### GO Enrichment of Differentially Expressed miRNAs, CircRNAs, lncRNAs and mRNAs in 4-NP-Treated Rat Primary Sertoli Cells *in vitro*

We determined the functional categories of differentially expressed miRNAs, circRNAs, lncRNAs and mRNAs between 4-NP-treated rat primary sertoli cells (0.5 mM, *in vitro*) and without 4-NP treatment *via* GO analysis to further understand the biological processes that may be mediated by these differentially expressed molecules in 4-NP-treated cells. For differentially expressed miRNAs, 827 GO terms in the category of biological processes were significantly enriched.

**FIGURE 4:**
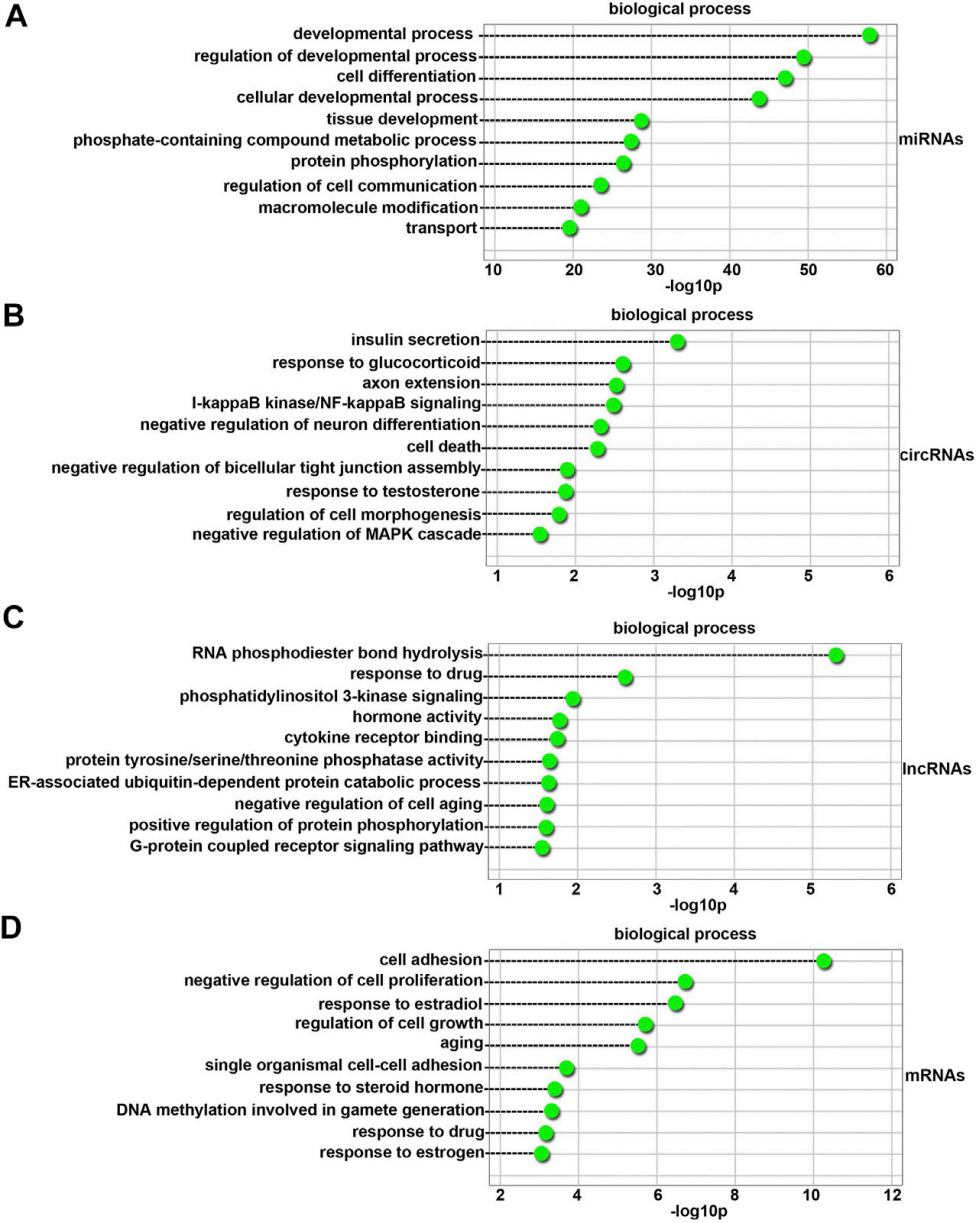
GO biological process analysis of differentially expressed miRNAs **(A)**, circRNAs **(B)**, lncRNAs **(C),** and mRNAs **(D)** between 4-NP-treated rat primary sertoli cells (0.5 mM, *in vitro*) and without 4-NP treatment. The dashed lines indicate the *p* values of the top 10 GO biological processes.

As shown in Figure 4A, the top 10 biological process terms with significant enrichment were the developmental process, regulation of developmental process, cell differentiation, cellular developmental process, tissue development, phosphate-containing compound metabolic process, protein phosphorylation, regulation of cell communication, macromolecule modification, and transport terms.

A total of 100 differentially expressed circRNAs were annotated with GO terms. The top 10 biological process terms with significant enrichment are shown in Figure 4B. They were the insulin secretion, response to glucocorticoid, axon extension, I-kappaB kinase/NF-kappaB signaling, negative regulation of neuron differentiation, cell death, negative regulation of bicellular tight junction assembly, response to testosterone, regulation of cell morphogenesis, and negative regulation of mitogen-activated protein kinases (MAPK) cascade terms. Many of the biological process terms were related to sertoli cell development, such as the response to testosterone and negative regulation of the MAPK cascade terms.

A total of 100 differentially expressed lncRNAs were annotated with GO terms. As shown in Figure 4C, the top 10 biological process terms with significant enrichment were the RNA phosphodiester bond hydrolysis, response to drug, phosphatidylinositol 3-kinase signaling, hormone activity, cytokine receptor binding, protein tyrosine/serine/threonine phosphatase activity, ER-associated ubiquitin-dependent protein catabolic process, negative regulation of cell aging, positive regulation of protein phosphorylation, and G-protein coupled receptor signaling pathway terms. The most abundant terms were those associated with the functions of sertoli cells.

There were also one hundred GO terms that were significantly enriched for differentially expressed genes. As shown in Figure 4D, the top 10 biological process terms with significant enrichment were the cell adhesion, negative regulation of cell proliferation, response to estradiol, regulation of cell growth, aging, single organismal cell-cell adhesion, response to steroid hormone, DNA methylation involved in gamete generation, response to drug, and response to estrogen terms. The most abundant terms were those associated with the functions of sertoli cells and the medicinal properties of 4-NP.

### KEGG Enrichment of Differentially Expressed miRNAs, circRNAs, lncRNAs and mRNAs in 4-NP-Treated Rat Primary Sertoli Cells *in vitro*

**FIGURE 5:**
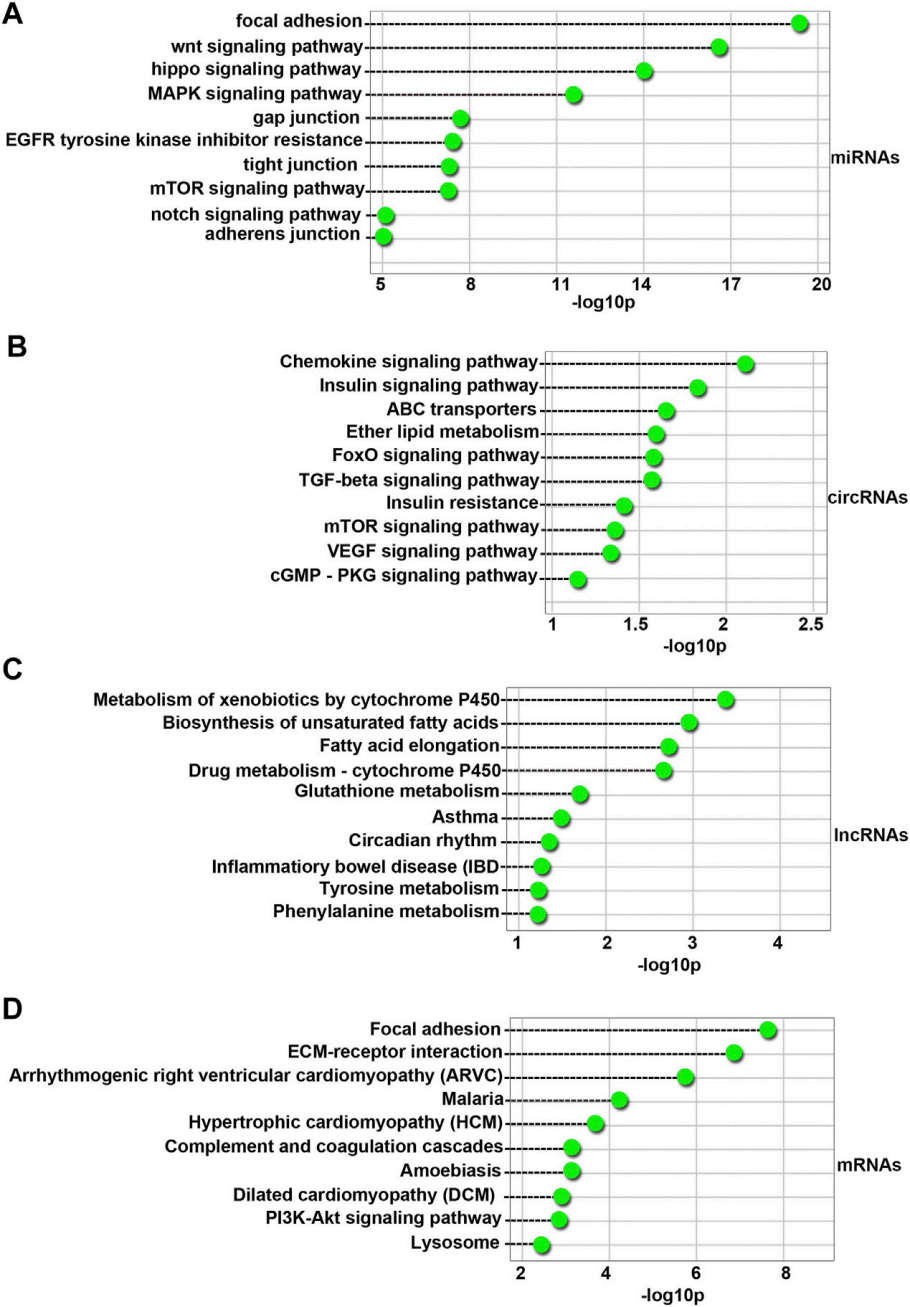
Top 10 enriched KEGG pathways for the differentially expressed miRNAs **(A)**, circRNAs **(B)**, lncRNAs **(C)**, and mRNAs **(D)** between 4-NP-treated rat primary sertoli cells (0.5 mM, *in vitro*) and without 4-NP treatment. The dashed lines represent the *p* values of the top Ten significantly enriched KEGG pathways.

One hundred fifty-six KEGG pathways (FDR < 0.05) were associated with differentially expressed miRNAs. As shown in Figure 5A, the top 10 enriched pathway terms were the Focal adhesion, Wnt signaling pathway, Hippo signaling pathway, MAPK signaling pathway, Gap junction, EGFR tyrosine kinase inhibitor resistance, tight junction, mTOR signaling pathway, Notch signaling pathway, and Adherens junction terms. These results indicated that 4-NP injured the blood-testis barrier (BTB) in sertoli cells *in vitro*.

Sixteen KEGG pathways (FDR < 0.05) were associated with host genes of differentially expressed circRNAs. As shown in Figure 5B, the top Ten enriched pathway terms were the Chemokine signaling pathway, Insulin signaling pathway, ABC transporters, Ether lipid metabolism, FoxO signaling pathway, TGF-beta signaling pathway, Insulin resistance, mTOR signaling pathway, VEGF signaling pathway, and cGMP-PKG signaling pathway terms.

Seven KEGG pathways (FDR < 0.05) were associated with differentially expressed lncRNAs. As shown in Figure 5C, the top 10 enriched pathway terms were the Metabolism of xenobiotics by cytochrome P450, Biosynthesis of unsaturated fatty acids, Fatty acid elongation, Drug metabolism - cytochrome P450, Glutathione metabolism, Asthma, Circadian rhythm, Inflammatory bowel disease (IBD), Tyrosine metabolism, and Phenylalanine metabolism terms.

Fifty-one KEGG pathways (FDR < 0.05) were associated with differentially expressed mRNAs. As shown in Figure 5D, the top 10 enriched pathway terms were the Focal adhesion, ECM-receptor interaction, Arrhythmogenic right ventricular cardiomyopathy (ARVC), Malaria, Hypertrophic cardiomyopathy (HCM), Complement and coagulation cascades, Amebiasis, Dilated cardiomyopathy (DCM), PI3K-Akt signaling pathway, and Lysosome terms.

### Validation of circRNAs and mRNAs Expression in 4-NP-Treated in 4-NP-Treated Rat Primary Sertoli Cells *in vitro*

**FIGURE 6:**
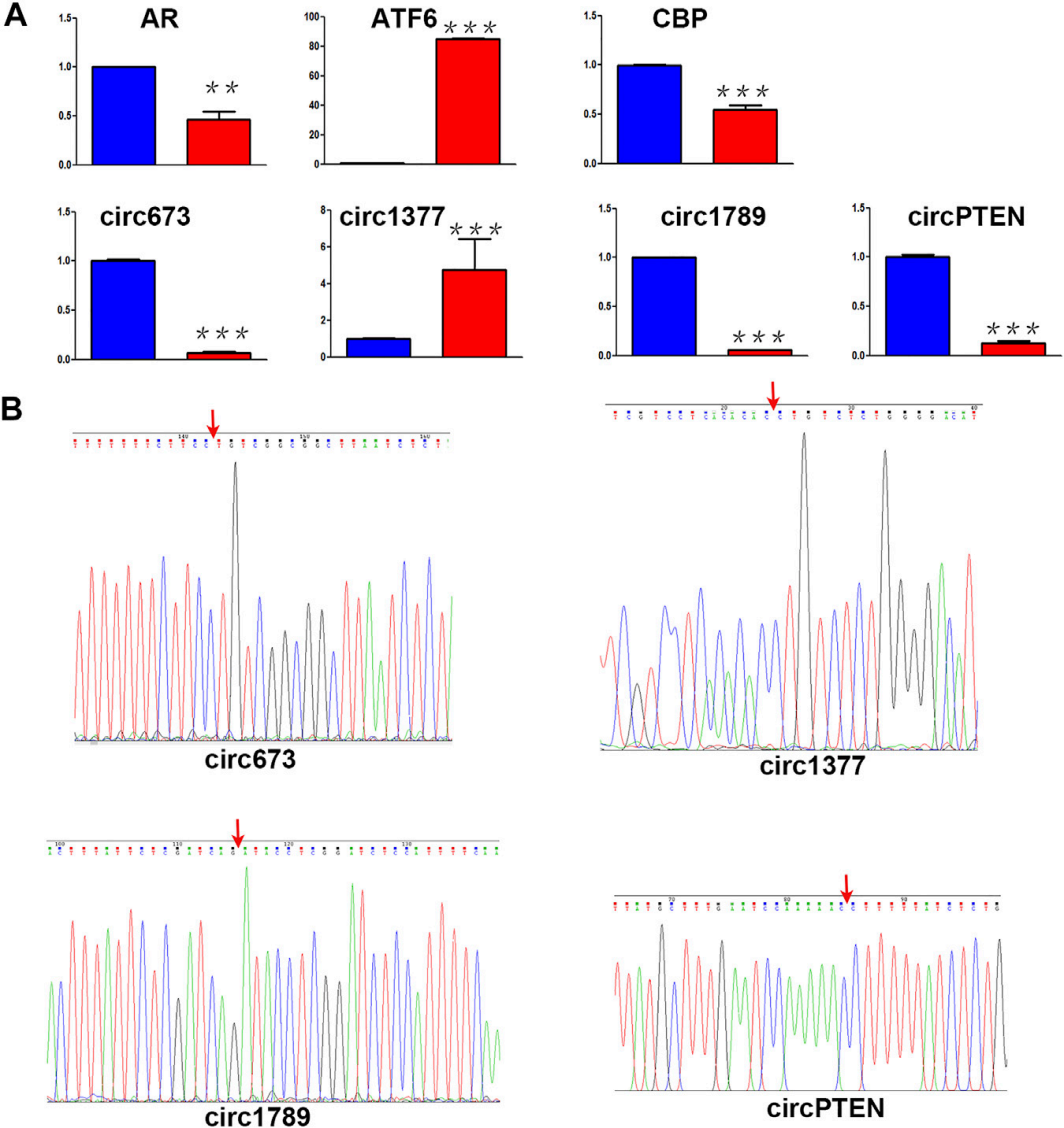
Identification of ceRNA **(A)** Differential RNA expression between 4-NP-treated rat primary sertoli cells (Red, 0.5 mM, *in vitro*) and without 4-NP treatment (blue) was validated by RT-qPCR **(B)** the head-to-tail splicing of *circ673*, *circ1377*, *circ1789*, and *circPTEN* was identified by Sanger sequencing.

A core set of mRNAs (*Ar*, *Atf6* and *Cbp*) and circRNAs (*circ673*, *circ1377*, *circ1789* and *circPTEN*) identified as differentially expressed by ceRNA/miRNA-Seq (Figure 6A) were validated by real‐time quantitative polymerase chain reaction (RT‐qPCR). As shown in Figure 6A, the RT-qPCR results were highly consistent with the RNA-sequence data. In addition, we performed sanger sequencing of RT-qPCR products in PMD-19T plasmids, and the results shown in Figure 6B confirmed that the products were *circ673*, *circ1377*, circ*1789* and *circPTEN*. Melting curve analysis and agarose gel electrophoresis were used to check for the specificity of the RT-qPCR products.

### Global ceRNA Network Integration

**FIGURE 7:**
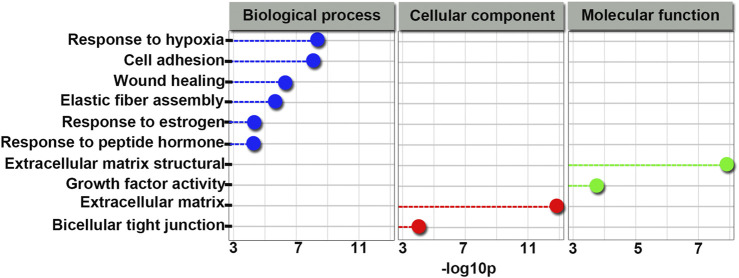
GO terms related to the global integrated ceRNA network involved in the effect of 4-NP on rat sertoli cells. Blue indicates biological processes; green indicates molecular functions and red indicates cellular components.

Based on our sequencing data, we used Cytoscape_V2_8_3 to predict a global ceRNA network. As shown in Supplementary Table S2 and Figure 7, we conducted further characterization of circRNA-associated ceRNA networks and performed GO analysis of the biological processes. Notably, we observed that the target genes of this module were related to biological processes and molecular functions of sertoli cells, such as cell adhesion and bicellular tight junction formation. In addition, we selected *circ673*, *circ1377*, *circ1789*, and *circPTEN* from the global ceRNA network and predicted their ceRNA interactions based on our RNA-Seq data by using Cytoscape_V2_8_3. As shown in Supplementary Table S3, both *circ673* and *circ1789* were predicted to regulate the expression of AR through ceRNA interactions. Both *circ1377* and *circPTEN* were predicted to regulate the expression of ATF6 through ceRNA interactions.

## Discussion and Perspectives

It is well demonstrated that estrogen plays an important role in the maintenance of male reproduction and homeostasis under physiological Pentikainen et al. (2000) and pathological conditions Chaki et al. (2006) ^.^ Xenoestrogens such as NPs are considered endocrine-disrupting chemicals (EDCs) to which humans are commonly exposed in daily life because of their widespread presence. NPs pose health risks predominantly because of their estrogenic-like effects in interactions with male reproductive systems. For instance, NPs reduce sperm viability and fertility in mature male ducks (Cheng et al., 2016). NPs have adverse effects on testicular structure in male fish Christiansen et al. (1998) and on tight junctions of sertoli cells in male rats (Hu et al., 2014). It has been reported that NPs induce apoptosis Choi et al. (2014) and necrosis Duan et al. (2016) in sertoli cells *via* reactive oxygen species (ROS) generation. It has also been found that NPs enhance the expression of cell cycle control genes in human prostate nontumorigenic cells, which suggests that other pathways may be associated with NP action on prostates (Forte et al., 2016). However, a report have shown that the potential endocrine hazard posed by NPs to humans is likely to be negligible (Moffat et al., 2001).

It has been reported that NPs are widely distributed in the environment, and small amounts of NPs can leach out into the food chain and drinking water Soares et al. (2008), which makes human exposure unavoidable and most likely lifelong. A previous study by the Centers for disease Control and Prevention (CDC) detected NPs in the urine of > 50% of Americans sampled in 2005 (Calafat et al., 2005). In addition, urinary NPs levels have been shown to be associated with increased early maturation and health implications in pubertal girls and with male infertility (Chen et al., 2013).

AR plays crucial roles in the maturation and function of the male reproductive system (Chang et al., 2013). The adverse impacts of chemical substances on male reproductive function are often associated with AR abnormalities, such as changes in AR expression, blocked AR signaling and reduced AR activity (Mylchreest et al., 1999; Manfo et al., 2014). *In vivo* experiments have shown that sertoli cell-specific AR-knockout mice (S-AR (-/y) mice) are infertile, and almost no sperm are detected in the epididymides (Chang et al., 2004). It has been reported that NPs act as AR antagonists Xu et al. (2005) and that the mRNA expression levels of androgen receptor (AR) are downregulated with NP exposure in sertoli TM4 cells (Liu et al., 2014). It has also been found that xenoestrogens with antiandrogenic functions prevent conformational change by stabilizing complexes with associated proteins (Kuil et al., 1995).

Xenoestrogens such as BPA have been reported to contribute to obvious gene expression in prostate cancer cells with somatic AR mutations (Hess-Wilson et al., 2007). It has also been found that NPs induce apoptosis and DNA damage through activation of PARP, Caspase-3, and Histone H2A.X (Kim et al., 2018).

CircRNAs and lncRNAs can act as sponges for miRNAs and participate in a variety of important biological processes (Kumar et al., 2017; Hu et al., 2018; Hu et al., 2019; Ishola et al., 2020). Spermatogenesis is dependent on sertoli cells, whose main function is to provide support and nutrition for spermatogenesis (Riera et al., 2003; Jan et al., 2012). At present, there are few reports about the type, quantity, functions and regulation mechanisms of ceRNAs in sertoli cells of the testis (Qin et al., 2019).

Characterization of ceRNA networks is a new way to discover the underlying mechanisms of the effects of NPs on sertoli cells. However, ceRNA signatures have been poorly examined in NP-treated sertoli cells until now. Therefore, the expression profiles of circRNAs, lncRNAs, mRNAs and miRNAs in NP-treated sertoli cells and controls were detected and analyzed in our study. This study provides a new experimental basis for exploring the role of ceRNAs in the impairment of sertoli cell function induced by NPs. In this study, primary sertoli cells were isolated and treated with a 4-NP or a control. Total RNA was extracted from the samples and then processed to prepare whole libraries. The roles of ceRNAs in the damage of sertoli cell function induced by 4-NP were studied by using Illumina HiSeq sequencing and analysis, bioinformatics, GO and KEGG pathway analysis, and RT-qPCR. In this study, we identified 54 upregulated and 82 downregulated miRNAs, 465 upregulated and 419 downregulated circRNAs, 74 upregulated and 191 downregulated lncRNAs, and 1,747 upregulated and 2,659 downregulated mRNAs. The functional annotations and pathway analyses showed that the most abundant categories were those associated with the functions of sertoli cells and the medicinal properties of NPs. Moreover, a global ceRNA network was also analyzed in this study. Head-to-tail splicing of the RT-qPCR products for *circ673*, *circ1377*, *circ1789* and *circPTEN* was confirmed by Sanger sequencing. The results provide new insights for elucidating the roles of ceRNAs in the functional impairment of NP-exposed sertoli cells and provide data regarding the molecular mechanisms by which ceRNAs participate in the functional impairment of NP-exposed sertoli cells.

In this work, we evaluated reproductive impairment of xenoestrogens 4-nonylphenols (4-NPs) and provided the first insight into the ceRNA expression profiles of rat sertoli cells, which reveal that ceRNAs participate in 4-NP-induced impairment of sertoli cell function, thereby indicating potential therapies for both reproductive toxicology and male infertility. These data improve understanding of the roles of xenoestrogens in male infertility. Further investigations are required to establish the biochemical and molecular bases for reproductive disorders.

## Data Availability

The datasets presented in this study can be found in online repositories. The names of the repository/repositories and accession number(s) can be found below: NCBI Sequence Read Archive under accession number GSE167912.

## Supplementary Material

Supplementary Figure S1 | Analysis of the miRNA-Seq data. **(A)** Volcano plot of the differentially expressed miRNAs. **(B)** A total of 136 differentially expressed miRNAs were identified, including 54 upregulated and 82 downregulated miRNAs. **(C)** Correlation analysis of all samples. **(D)** Length distribution of sRNAs in six samples.

Supplementary Figure S2 | Analysis of the circRNA-Seq data. **(A)** Volcano plot of the differentially expressed circRNAs. **(B)** A total of 884 differentially expressed circRNAs were identified, including 465 upregulated and 419 downregulated circRNAs. **(C)** The distribution of circRNA-Seq data for each group was analyzed, and the data are shown in a box plot. **(D)** circRNA-Seq quality control.

Supplementary Figure S3 | Analysis of the lncRNA-Seq data. **(A)** Volcano plot of the differentially expressed lncRNAs. **(B)** A total of 265 differentially expressed lncRNAs were identified, including 74 upregulated and 191 downregulated lncRNAs. **(C)** The distribution of lncRNA-Seq data for each group was analyzed, and the data are shown in a box plot. **(D)** Correlation analysis of all samples. **(E)** lncRNA-Seq quality control.

Supplementary Figure S4 | Analysis of the mRNA-Seq data. **(A)** Volcano plot of the differentially expressed genes. **(B)** A total of 4,406 differentially expressed genes were identified, including 1,747 upregulated and 2,659 downregulated genes. **(C)** The distribution of mRNA-Seq data for each group was analyzed, and the results are shown in a box plot. **(D)** The precision of any sample statistic (RPKM) analysis of mRNA-Seq experiments.

Supplementary Table S1 | The primer pairs (circRNAs and mRNAs) for quantitative PCR.

Supplementary Table S2 | global ceRNA networks integration in 4-NPs vs controls.

Supplementary Table S3 | ceRNA networks of circ673, circ1377, circ1789, and circPTEN in 4-NPs vs controls.
