# Supplementary figures and images for: Identification of Competing Endogenous RNA and Micro-RNA Profiles and Regulatory Networks in 4-Nonylphenol-induced Impairment of Sertoli Cells
Source: Front Pharmacol. 2021 May 18;12:644204. doi: 10.3389/fphar.2021.644204 (PMC8167654; doi:10.3389/fphar.2021.644204)

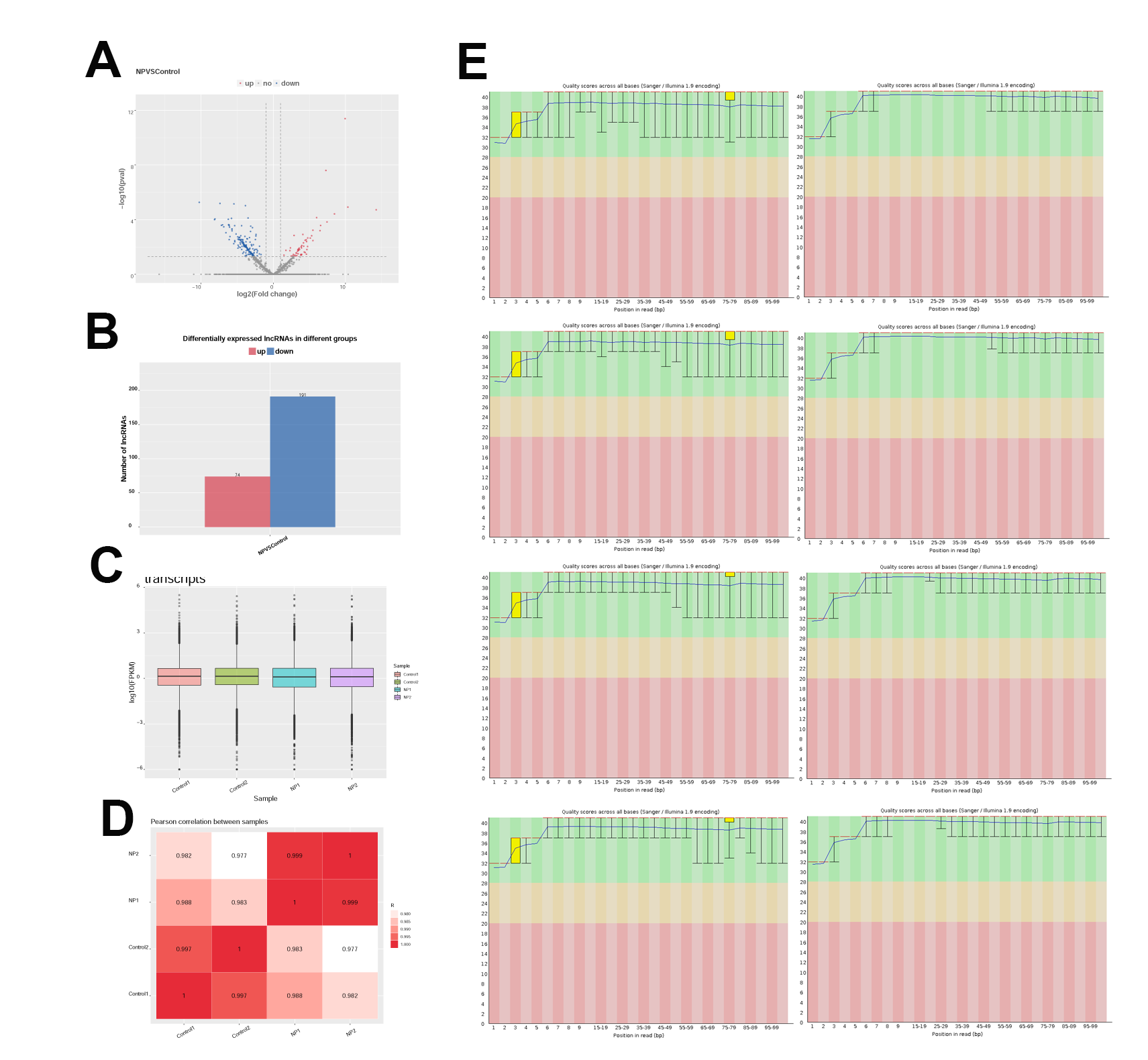

Supplement: Supplementary file 4 [file image3.tif]

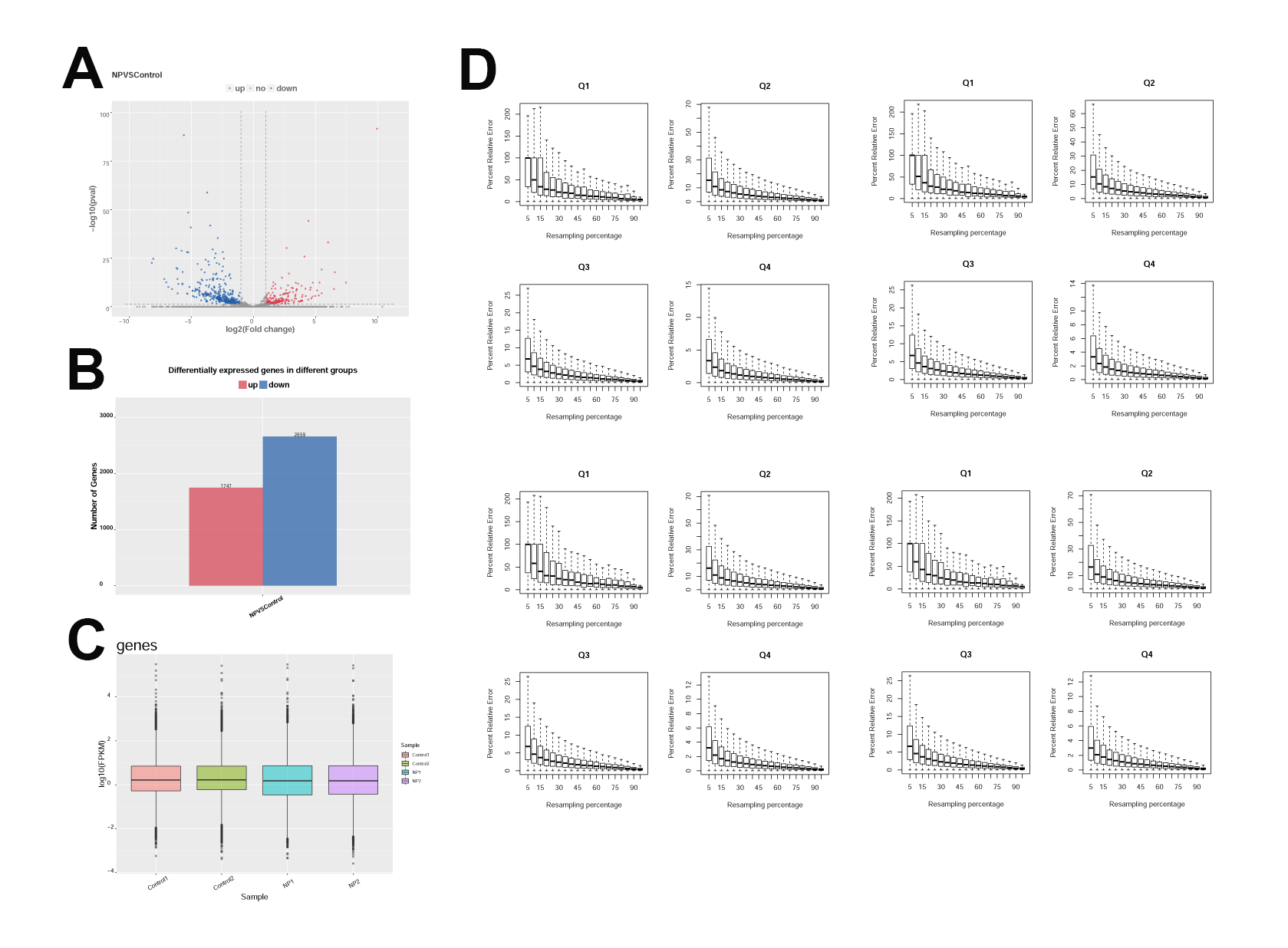

Supplement: Supplementary file 5 [file image4.tif]

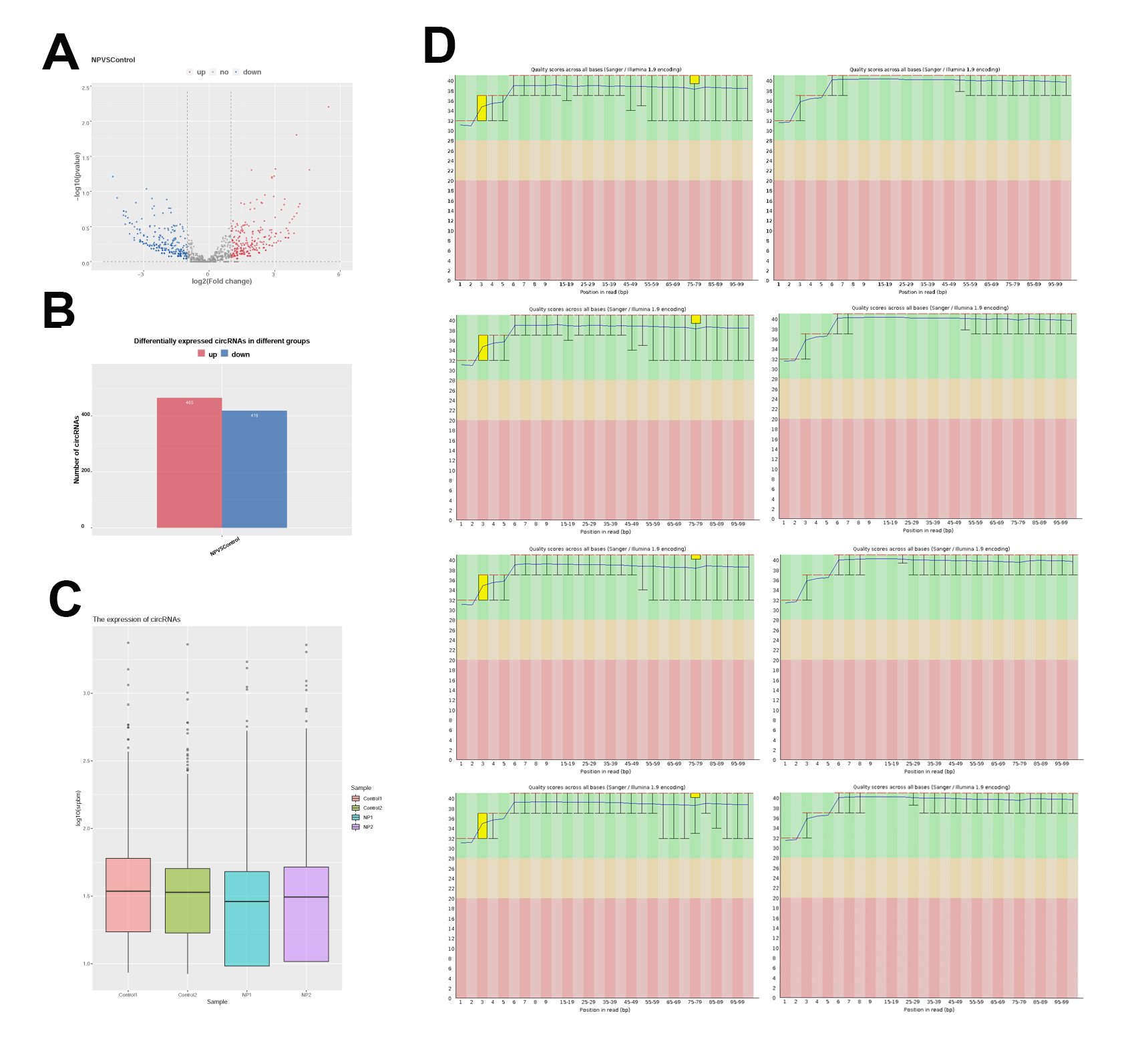

Supplement: Supplementary file 6 [file image2.tif]

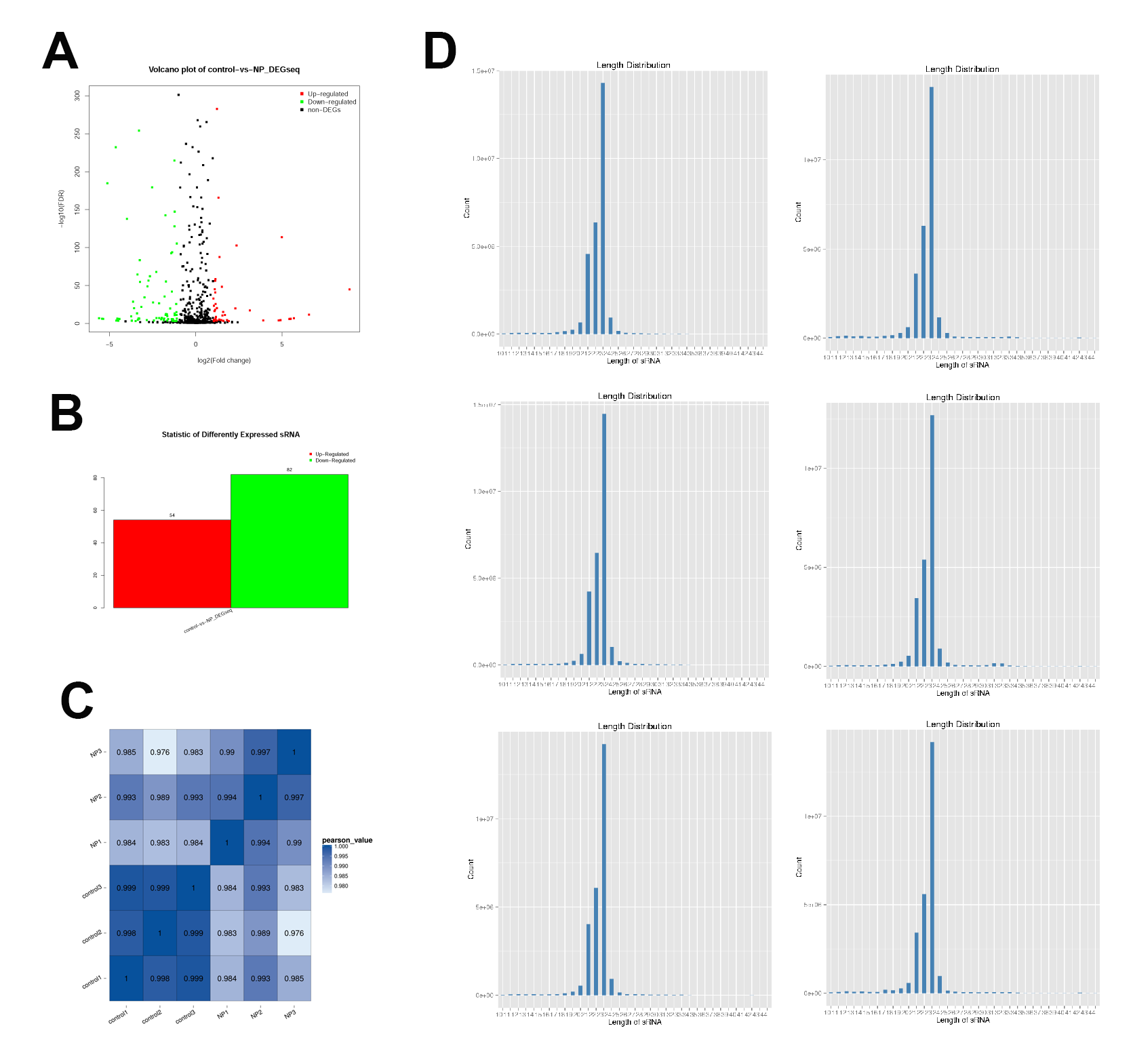

Supplement: Supplementary file 7 [file image1.tif]
